# Reverse Cardiac and Epicardial Adipose Tissue Remodeling Following Catheter Ablation of Paroxysmal Atrial Fibrillation in HFpEF

**DOI:** 10.3390/diagnostics16162492

**Published:** 2026-08-07

**Authors:** Jan Alatič, David Šuran, Husam Franjo Naji, Maja Pirnat

**Affiliations:** 1 University Department of Cardiology and Angiology, University Medical Centre Maribor, Ljubljanska Ulica 5, 2000 Maribor, Slovenia; david.suran@ukc-mb.si (D.Š.); husamfranjo.naji@ukc-mb.si (H.F.N.); 2 Faculty of Medicine, University of Maribor, Taborska Ulica 8, 2000 Maribor, Slovenia; 3 Department of Radiology, University Medical Centre Maribor, Ljubljanska Ulica 5, 2000 Maribor, Slovenia

**Keywords:** paroxysmal atrial fibrillation, catheter ablation, HFpEF, reverse remodeling, cardiac magnetic resonance imaging, epicardial adipose tissue

## Abstract

**Background**: Catheter ablation (CA) with pulmonary vein isolation is an established treatment for atrial fibrillation (AF). Epicardial adipose tissue (EAT) has been implicated in AF pathophysiology, but data on periatrial EAT and cardiac structural changes after CA in patients with heart failure with preserved ejection fraction (HFpEF) remain limited. **Methods**: We prospectively included 43 patients with paroxysmal AF and HFpEF undergoing radiofrequency CA. Cardiac magnetic resonance (CMR) was performed before ablation and after 6 months to quantify periatrial EAT volume and left ventricular mass (LVM) as prespecified primary endpoints, alongside secondary exploratory structural and functional parameters. **Results**: The study included 43 patients (74.4% male; mean age 60.5 ± 10.4 years). At 6-month follow-up, both primary endpoints improved significantly: periatrial EAT volume decreased from 28.8 ± 6.3 to 20.3 ± 5.9 mL (*p* < 0.001), and LVM decreased from 102 (95–115) to 97 (89–104) g/m^2^ (*p* = 0.003). Exploratory analyses demonstrated favorable reverse cardiac remodeling, including reductions in left atrial area (LAA), left ventricular end-diastolic and end-systolic volumes, right ventricular end-systolic volume, and an increase in left ventricular stroke volume. Greater reduction in periatrial EAT volume correlated with more pronounced reverse cardiac remodeling, particularly with reductions in LVM (ρ = 0.48; *p* < 0.001) and LAA (ρ = 0.44; *p* = 0.003), with similar associations observed across other left ventricular remodeling parameters. **Conclusions**: In patients with paroxysmal AF and HFpEF, CA was associated with significant reductions in periatrial EAT volume and LVM, accompanied by favorable structural changes on CMR. These findings are consistent with reverse cardiac remodeling following CA and support further investigation of its structural effects in this patient population.

## 1. Introduction

Atrial fibrillation (AF) is the most common sustained cardiac arrhythmia and remains a growing global health burden [1,2,3,4]. Its prevalence continues to rise, largely driven by population aging and the increasing burden of cardiovascular and metabolic comorbidities [4,5]. AF is not a static condition but a progressive process shaped by a complex interplay of inflammation, myocardial fibrosis, oxidative stress, and structural as well as electrical remodeling of the atria [3,4].

In recent years, increasing attention has focused on the role of obesity and visceral adiposity in AF pathophysiology. Among these, epicardial adipose tissue (EAT) has emerged as a key player. Positioned in direct contact with the myocardium, EAT is a metabolically active tissue capable of exerting both protective and harmful effects [3,4,6,7,8,9]. Under pathological conditions, its expansion promotes local inflammation, fibrosis, and atrial remodeling, and has been consistently linked to both AF development and recurrence after catheter ablation (CA) [10,11,12].

CA with pulmonary vein isolation (PVI) is a well-established treatment for symptomatic AF that improves quality of life [10,11,12]. At the same time, the way we define its success is evolving, shifting from a simple binary assessment of AF recurrence toward AF burden as a more clinically meaningful measure [13,14]. Although many landmark trials, including CABANA, have primarily relied on binary definitions of ablation success, studies incorporating continuous rhythm monitoring, such as CASTLE-AF, have demonstrated that a reduction in AF burden is associated with improved clinical outcomes, particularly in patients with heart failure with reduced ejection fraction (HFrEF) [13,14,15,16,17,18].

However, the interplay between AF burden, EAT, and cardiac remodeling in patients with heart failure with preserved ejection fraction (HFpEF) remains poorly understood. While HFrEF is primarily characterized by impaired left ventricular (LV) systolic function resulting from myocardial injury, LV remodeling, and neurohormonal activation, HFpEF represents a heterogeneous syndrome driven by inflammation, structural, and metabolic dysfunction [15,16,17,19,20,21,22,23,24]. Although AF is highly prevalent in both conditions, the underlying arrhythmogenic substrate differs substantially. In HFrEF, AF predominantly develops secondary to hemodynamic stress, LV dysfunction, chamber dilatation, increased filling pressures, and secondary atrial remodeling. In contrast, AF in HFpEF is largely promoted by systemic comorbidities, chronic low-grade inflammation, atrial myopathy, and expansion of EAT, leading to left atrial inflammation, increased stiffness, and progressive atrial fibrosis [15,16,17,19,20,21,22,23,24].

In this context, the aim of the present study was to evaluate the association between radiofrequency CA and changes in cardiac morphology, function, and periatrial EAT volume in patients with HFpEF and paroxysmal AF, using cardiac magnetic resonance imaging (CMR), the reference standard for cardiac volumetric assessment.

## 2. Materials and Methods

We prospectively included 43 patients with paroxysmal AF and established HFpEF who underwent radiofrequency CA with PVI. HFpEF diagnosis was established prior to study enrollment in accordance with current ESC guidelines as a LV ejection fraction (LVEF) ≥ 50%, presence of signs and/or symptoms of heart failure, and objective evidence of diastolic dysfunction on baseline echocardiography and/or elevated natriuretic peptide levels [25]. The H_2_FPEF score was additionally used to support and confirm the diagnosis of HFpEF. Baseline transthoracic echocardiography was performed in patients with a stable sinus rhythm. Blood samples (including NT-proBNP) were collected upon admission for CA, also during a verified sinus rhythm.

Patients with an LVEF lower than 50%, known ischemic heart disease, infiltrative, hypertrophic cardiomyopathy, or moderate-to-severe valvular stenosis or regurgitation were excluded from the study. Before the procedure, a detailed patient history was obtained, including age, body weight, body mass index (BMI), comorbidities, and the type of antiarrhythmic therapy. Absence of diabetes mellitus was defined as HbA1c < 6.5% at admission and no prior diagnosis of diabetes mellitus or use of antidiabetic therapy. Absence of ischemic heart disease was defined as the absence of previous cardiac events, angina, or wall motion abnormalities on echocardiography. Absence of chronic pulmonary disease was defined by detailed history and the absence of concomitant inhalatory pharmacotherapy or non-invasive inhalatory therapy.

One day prior to ablation, CMR was performed in all patients to evaluate cardiac morphology, function, and EAT of the left atrium. All measurements were done by a single experienced radiologist. Radiofrequency CA with PVI was done the day after, also by a single experienced operator. Patients were followed for 6 months, after which CMR was repeated.

An a priori power analysis was performed to determine the required sample size. Based on the expected change in LV mass (LVM) index reported by Rattka et al., a minimum of 16 patients was required to detect a clinically significant difference in LVM, assuming a two-sided α level of 0.05 and power of 90% [26]. Written informed consent was obtained from all participants prior to inclusion in the study. The study was conducted in accordance with the principles of the Declaration of Helsinki and was approved by the National Medical Ethics Committee (0120-445/2021-2711-6).

### 2.1. CMR Imaging

All patients underwent CMR using a 1.5-T scanner (MAGNETOM Sola, Siemens Healthineers, Forchheim, Germany). All CMR image acquisitions were performed while patients were in a stable sinus rhythm. Cardiac morphology and function were assessed using cine balanced steady-state free precession (bSSFP) sequences acquired in standard long-axis views (two-chamber, three-chamber, and four-chamber) and contiguous short-axis slices covering the entire ventricles from base to apex.

Using dedicated post-processing software (cvi42, version: 6.3.1 (4801), Circle Cardiovascular Imaging, Calgary, AB, Canada), the following parameters were quantified: LVEF, LV end-diastolic volume (LVEDV), LV end-systolic volume (LVESV), LV stroke volume (LVSV), LVM, right ventricular end-diastolic volume (RVEDV), right ventricular end-systolic volume (RVESV), right ventricular stroke volume (RVSV), left atrial area (LAA), and right atrial area (RAA). Periatrial EAT volume was evaluated and quantified using bSSFP cine images at end-diastole with the help of syngo.via software (version VB60, Siemens Healthineers, Forchheim, Germany). It was defined as the adipose tissue localized between the outer surface of the atrial myocardium and the visceral layer of the pericardium. The periatrial region was segmented and manually contoured on a slice-by-slice basis, with the superior limit defined at the level of the pulmonary artery bifurcation and the inferior limit defined at the atrioventricular groove. Periatrial EAT volume was therefore obtained using signal intensity thresholding and software-integrated volumetric calculations.

### 2.2. Radiofrequency CA with PVI

All procedures were done in conscious sedation. Under fluoroscopic guidance, a transseptal puncture was performed. A detailed bipolar voltage map of the left atrium was then constructed using a 20-polar catheter (Pentaray; Biosense-Webster, Irvine, CA, USA). An automated 3D mapping system (Carto, Biosense Webster, Irvine, CA, USA) was used in all patients. Ablation was performed with a 3.5 mm irrigated-tipped catheter (SmartTouch Thermocool, Biosense Webster, Irvine, CA, USA). PVI was achieved with wide antral circumferential ablation. The isolation of the ablated region was then confirmed with entrance- and exit-block pacing maneuvers, as well as post-ablation LA voltage mapping. Direct current cardioversion to restore sinus rhythm was also performed after the successful procedure if patients were still in AF [27].

### 2.3. Follow-Up

All patients had a 12-lead ECG (25 mm/s, 10 mm/mV) recorded at 1, 2, and 6 months after CA to evaluate their basic rhythm. After 6 months, all patients underwent 24 h Holter monitoring as well. Patients were also instructed to visit an outpatient clinic earlier if symptoms suggested AF recurrence. All patients underwent CMR evaluation 6 months after CA in order to establish the impact of CA on cardiac function, morphology, and periatrial EAT volume.

### 2.4. Statistical Analysis

Statistical analyses were performed using SPSS version 29 (SPSS Inc., Chicago, IL, USA). Normality of the distribution was assessed using the Kolmogorov–Smirnov test and by visual inspection of histograms. Continuous variables are presented as mean ± standard deviation for normally distributed data and as median (25th percentile–75th percentile) for non-normally distributed data. Categorical variables are expressed as counts and percentages. Given the longitudinal study design with repeated measurements in the same individuals, paired comparisons between baseline and the 6-month follow-up were performed. For normally distributed variables, differences were analyzed using the paired Student’s *t*-test, whereas the Wilcoxon signed-rank test was applied for non-normally distributed variables. All tests were two-tailed, and a *p*-value ≤ 0.05 was considered statistically significant. To account for multiple comparisons, the analysis was organized around pre-specified primary and exploratory endpoints. Periatrial EAT and LVM were defined as the primary endpoints, while LAA, LVEDV, LVESV, LVSV, RAA, RVEDV, RVESV, and RVSV were treated as exploratory endpoints. To control the false discovery rate among the exploratory analyses, the Benjamini–Hochberg procedure was applied separately to the sets of exploratory endpoints for the left and right cardiac chambers. A q-value (adjusted *p*-value) of <0.05 was considered statistically significant. Analyses were performed on a complete-case basis. Furthermore, changes in periatrial EAT and cardiac structural parameters were evaluated for correlation using the non-parametric Spearman’s rank correlation test due to the non-normal distribution of the data.

## 3. Results

In our prospective study, we included 43 patients with paroxysmal AF, predominantly male (74.4%), with an average age of 60.51 ± 10.43 years. All patients met the diagnostic criteria for HFpEF prior to inclusion, as each patient fulfilled the ESC guideline echocardiographic criteria and had an H_2_FPEF score ≥ 6 (Table 1) [25]. Before enrollment, all patients were clinically symptomatic for heart failure. Antiarrhythmic therapy was continued in all patients after the CA, with propafenone alone or combined with a beta-blocker (in 65.1% of cases) being the only agents used. The AF recurrence rate was 26.9% at 6 months of follow-up, recorded by either Holter ECG monitoring or interim ECG controls. At admission, 91% of patients presented with elevated NT-proBNP levels exceeding the ESC guideline-recommended diagnostic threshold for chronic heart failure (NT-proBNP ≥ 125 pg/mL) (Table 1) [25]. Clinical characteristics, transthoracic echocardiographic data at enrollment, and laboratory results on admission are presented in Table 1.

### 3.1. Impact of CA of AF on Cardiac Remodeling

**Figure 1:**
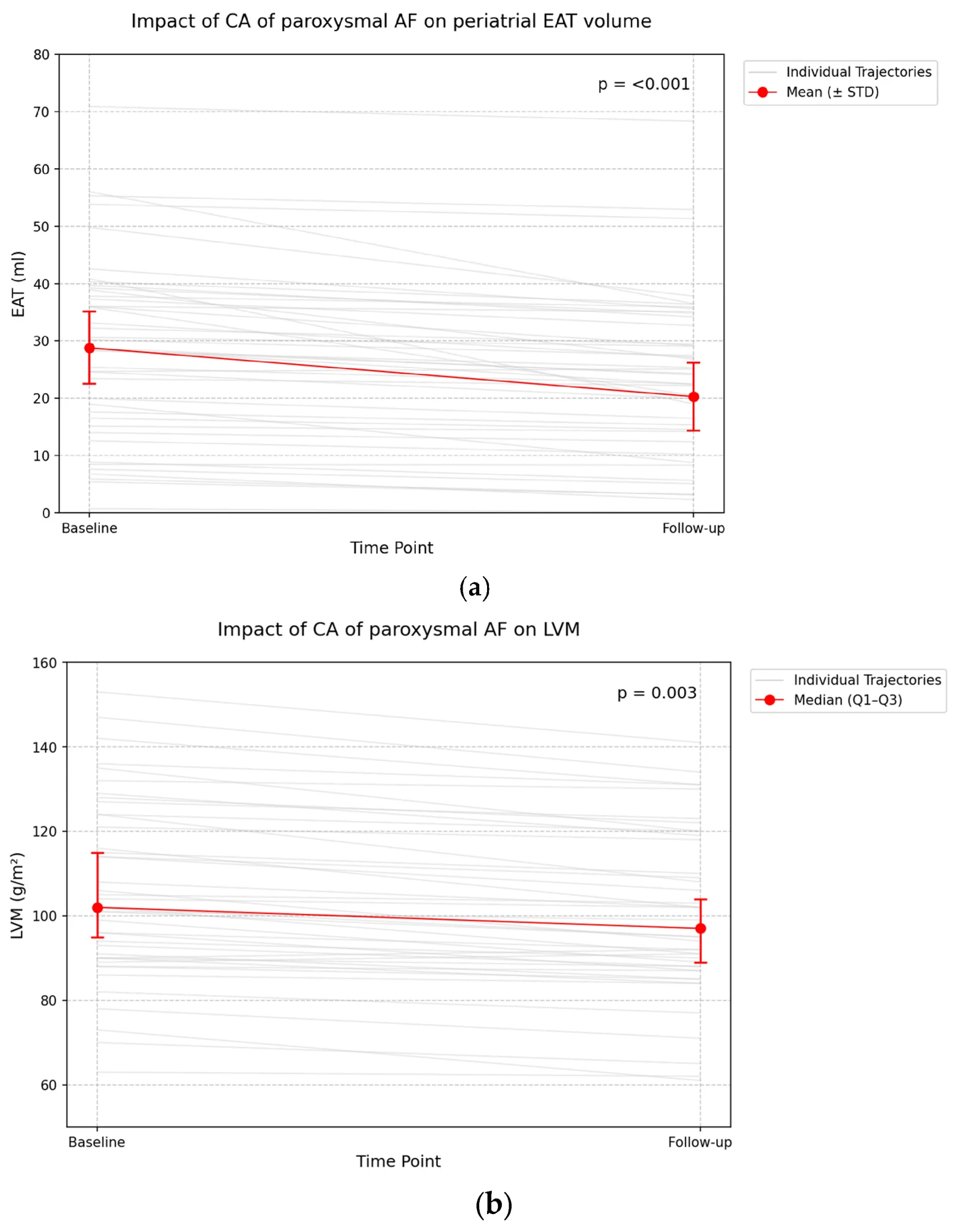
Impact of CA of paroxysmal AF on periatrial EAT volume (**a**) and LVM (**b**). CA—catheter ablation; AF—atrial fibrillation; EAT—epicardial adipose tissue; LVM—left ventricular mass.

All patients had a normal baseline LVEF (59.0 ± 3.2%). Periatrial EAT volume at 6 months after CA was significantly lower compared to the baseline atrial EAT volume (20.3 ± 5.9 mL vs. 28.8 ± 6.3 mL, respectively; *p* = <0.001) (Table 2) (Figure 1).

**Figure 2:**
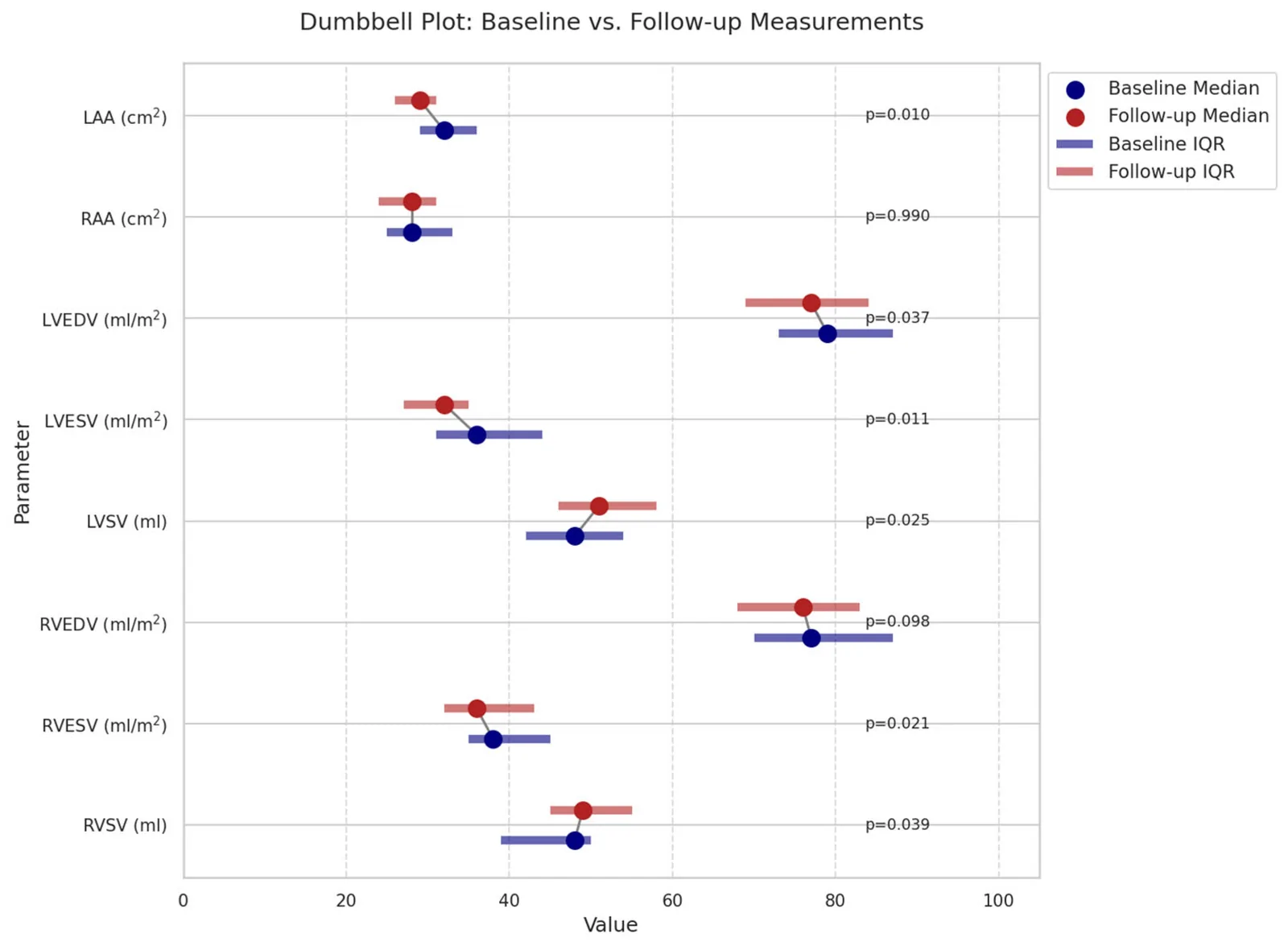
Impact of CA of paroxysmal AF on left and right heart chambers (exploratory endpoints). LAA—left atrial area; RAA—right atrial area; LVEDV—left ventricular end-diastolic volume; LVESV—left ventricular end-systolic volume; LVSV—left ventricular stroke volume; RVEDV—right ventricular end-diastolic volume; RVESV—right ventricular end-systolic volume; RVSV—right ventricular stroke volume.

LVM significantly decreased after 6 months after CA compared to the baseline evaluation prior to CA (97.0 (89–104) g/m^2^ vs. 102.0 (95–115) g/m^2^; *p* = 0.003) (Table 2) (Figure 1). At 6-month follow-up after CA we observed significantly lower LAA (29.0 (26–31) cm^2^ vs. 32.0 (29–36) cm^2^; *p* = 0.01), LVEDV (77.0 (69–84) mL/m^2^ vs. 79.0 (73–87) mL/m^2^; *p* = 0.037), LVESV (32.0 (27–35) mL/m^2^ vs. 36.0 (31–44) mL/m^2^; *p* = 0.011) and significantly increased LVSV (51.0 (46–58) mL vs. 48.0 (42–54) mL; *p* = 0.025) (Table 3) (Figure 2). Exploratory parameters remained significant even after Benjamini–Hochberg adjustment for multiple comparisons.

At 6-month follow-up after CA, RVESV was significantly decreased (36.0 (32–43) mL/m^2^ vs. 38.0 (35–45) mL/m^2^; *p* = 0.021) and RVSV was significantly increased (49.0 (45–55) mL vs. 48.0 (39–50) mL; *p* = 0.039), whereas RVEDV was lower but did not meet significantly relevant difference (76.0 (68–83) mL/m^2^ vs. 77.0 (70–87) mL/m^2^; *p* = 0.098). RAA did not change significantly (28.0 (24–31) cm^2^ vs. 28.0 (25–33) cm^2^; *p* = 0.99) (Table 3) (Figure 2). When Benjamini–Hochberg adjustment for multiple comparisons was applied, only the decrease in RVESV remained significant.

### 3.2. Correlations Between Periatrial EAT Reduction and Cardiac Remodeling

Correlation analyses demonstrated that greater reductions in periatrial EAT volume were consistently associated with more pronounced reverse LV remodeling. The strongest associations were observed for LVM (ρ = 0.48, *p* < 0.001) and LAA (ρ = 0.44, *p* = 0.003). Weaker but significant associations were also found for LVEDV, LVESV, and LVSV, whereas no significant correlations were observed for right-sided cardiac parameters (Table 4).

## 4. Discussion

In this prospective study, we included 43 relatively young patients with paroxysmal AF and HFpEF who underwent radiofrequency CA. After a successful CA at 6-month follow-up, we observed significant improvement in the primary endpoints—decreases in periatrial EAT volume and LVM. Besides the prespecified primary endpoints, exploratory analyses demonstrated additional favorable structural changes involving both left atrial and LV parameters, with a decrease in LAA, LVEDV, and LVESV and an increase in LVSV. Additionally, we observed a decrease in RVESV, with a nonsignificant trend toward improvement in RVEDV and RVSV. In the following section, the main findings are discussed.

### 4.1. EAT and AF

We demonstrated that periatrial EAT volume significantly decreased after CA across the entire cohort, independent of ablation outcome. Data evaluating the impact of CA of AF on EAT volume are scarce [28,29,30]. Our findings are consistent with two previous studies evaluating EAT remodeling after AF ablation. One study included only 15 patients, whereas the other demonstrated a significant reduction in EAT volume only in patients with persistent AF; in contrast, our cohort consisted exclusively of patients with paroxysmal AF [29,30].

The mechanisms underlying EAT volume reduction after CA remain incompletely understood and are likely multifactorial. In addition to direct thermal effects of CA and concomitant microvascular disruption of adjacent adipose tissue, sustained restoration of sinus rhythm may promote reverse atrial remodeling by improving atrial mechanical function, reducing atrial wall stress, attenuating neurohumoral activation, and suppressing local inflammatory signaling associated with AF [29,30,31]. Given the close anatomical and paracrine relationship between periatrial EAT and the adjacent atrial myocardium, improvement of the underlying atrial substrate may reduce inflammatory and metabolic stimuli that sustain EAT expansion [29,30,31]. Although these mechanisms remain hypothetical, they provide a biologically plausible explanation for the observed reduction in periatrial EAT volume after CA. Together, these changes may interrupt the proposed vicious cycle in which EAT promotes AF, which in turn promotes EAT [29,30].

Our study extends the limited existing evidence by evaluating periatrial EAT remodeling using CMR in a well-characterized cohort of patients with HFpEF and paroxysmal AF. Previous studies have demonstrated that EAT is associated with AF occurrence and represents an independent risk factor for AF recurrence after ablation [10,11,12]. Other studies investigating EAT remodeling after CA were conducted predominantly in mixed AF populations and primarily evaluated EAT remodeling according to binary ablation outcomes, namely AF recurrence or freedom from AF [3,7,11]. In contrast, we evaluated structural remodeling in the entire cohort irrespective of ablation outcome, acknowledging that CA may induce favorable cardiac remodeling even when complete freedom from AF is not achieved. This approach is consistent with the current understanding that AF represents a chronic progressive disease and that binary rhythm outcomes may not fully reflect the extent of structural remodeling following CA [13,14].

These findings may be particularly relevant in HFpEF because the pathophysiological substrate of AF differs substantially from that in HFrEF. Compared with HFrEF, AF in HFpEF is more closely linked to systemic inflammation, atrial cardiomyopathy, and periatrial EAT, where paroxysmal AF is more frequently encountered during earlier stages of atrial cardiomyopathy. This distinction may partly explain the favorable periatrial EAT remodeling observed in our cohort and suggests that periatrial EAT may represent a particularly relevant imaging biomarker and potential therapeutic target in HFpEF. Taken together, these pathophysiological differences underscore the need for dedicated studies in HFpEF rather than direct extrapolation of findings from HFrEF cohorts [15,16,17,18,19,20,21,22,23,24,32]. In this context, CMR imaging has emerged as a robust imaging modality and gained a significant role in tissue characterization and in detecting subclinical structural cardiac changes that cannot be reliably assessed by conventional methods, such as echocardiography [33,34].

### 4.2. Reverse Left Atrial and LV Remodeling

In parallel to the reduction in EAT volume, our results demonstrate that CA was also associated with favorable structural changes in both atrial and ventricular chambers. Specifically, we observed significant reductions in LAA, LVEDV, and LVESV, accompanied by an increase in LVSV. These findings, observed at the cohort level despite the presence of AF recurrence in a proportion of patients, suggest that complete arrhythmia elimination may not be necessary to achieve structural improvement, and that a reduction in AF burden is a plausible mechanism, although it was not directly quantified in the present study.

Mechanistically, lowering AF burden may reduce atrial wall stress, improve atrial contractility, and enhance ventricular filling, ultimately leading to improved diastolic function and stroke volume. In parallel, it may contribute to partial reversal of adverse electrical, neurohormonal, and inflammatory processes [35]. Our findings regarding structural improvement of the left atrium are consistent with previous studies, although these have largely focused on patients with HFrEF or persistent AF [19,21,35,36]. In contrast, data on reverse LV remodeling in HFpEF, particularly in patients with paroxysmal AF, remain limited [15,16,17,19,20,21,22]. What is more, most previous studies mainly used echocardiography to assess LV function and morphology, whereas CMR provides superior accuracy and reproducibility [26,37].

The observed reduction in LVM in our cohort may reflect reverse remodeling and could be related to attenuation of inflammatory and fibrotic processes. EAT, through its proinflammatory and profibrotic effects, has been linked to impaired diastolic function, and its reduction may therefore contribute to improved ventricular compliance [38]. This interpretation is supported by studies demonstrating subclinical myocardial changes associated with AF, as well as improvements in diastolic function following ablation in HFpEF populations [26,39].

Given the significant reductions and improvements in LVEDV, LVESV, and LVSV observed in our study, improvement in diastolic function may indeed represent a key mechanistic explanation for our findings. While reverse LV remodeling with improved prognosis and quality of life after AF ablation has been well described in patients with reduced LVEF [15,16,18,40], data in HFpEF—particularly in patients with paroxysmal AF—remain limited. In this context, our findings extend the current evidence by demonstrating reverse LV remodeling in this specific population.

### 4.3. Right Ventricular Remodeling

To the best of our knowledge, data on the impact of CA of AF on the right cardiac chambers is limited. We demonstrated that CA led to a significant reduction in RVESV, while RVEDV also decreased; however, the difference did not reach significance. RVSV increased following CA, although the change was non-significant. These findings suggest that CA exerts global beneficial effects on cardiac structure and has a favorable outcome in HFpEF patients with AF, potentially through improved ventricular interdependence, reduced pulmonary pressure secondary to improved left-sided filling dynamics, as well as a reduction in EAT volume.

### 4.4. Association Between Periatrial EAT Reduction and Reverse Cardiac Remodeling

Importantly, the extent of periatrial EAT reduction correlated with the magnitude of reverse cardiac remodeling. Greater reductions in periatrial EAT volume were associated with greater reductions in LVM and LAA, with similar associations observed across other LV remodeling parameters. These findings indicate that periatrial EAT remodeling occurs in parallel with structural cardiac recovery following CA. Although these correlations do not establish causality, they support the concept that periatrial EAT regression may represent an imaging marker of favorable reverse remodeling after restoration of sinus rhythm. Whether periatrial EAT actively contributes to reverse remodeling through attenuation of local inflammation or simply reflects broader myocardial recovery cannot be determined from the present study and remains hypothesis-generating [29,30,31].

To our knowledge, data examining the relationship between changes in periatrial EAT volume and comprehensive reverse cardiac remodeling assessed by CMR in patients with HFpEF remain scarce. Our findings therefore extend the existing evidence linking periatrial EAT to AF recurrence and atrial remodeling after CA by demonstrating significant associations between periatrial EAT regression and reverse remodeling across multiple left-sided cardiac structural parameters [29,30,31].

### 4.5. Strengths and Limitations

The main strength of our study is a relatively homogenous patient population, minimizing confounding comorbidities, such as ischemic heart disease or overt diabetes mellitus. Additionally, antiarrhythmic therapy was standardized (propafenone alone or combined with a beta-blocker), further reducing treatment variability. Importantly, CMR as an imaging modality was used for structural and functional assessment, serving as the gold standard for cardiac morphology and tissue characterization. CA and CMR analyses were performed by a single experienced operator and radiologist, respectively, thereby reducing interobserver variability.

Several limitations should also be acknowledged. First, the principal limitation of our study is the lack of systematic quantification of AF burden, as rhythm monitoring was limited to clinical assessment, ECG, and a single 24 h Holter recording. Consequently, AF burden may have been underestimated, and its relationship with LV remodeling could not be fully assessed. Nevertheless, our interpretation is supported by prior studies using continuous rhythm monitoring, which have consistently shown a significant reduction in AF burden following CA (e.g., CASTLE-AF, EARLY-AF, CABANA) [13,15,16]. Continuous monitoring strategies, such as implantable loop recorders, would allow more precise quantification of AF burden and a better understanding of its association with remodeling. Second, although the study exceeded the predefined sample size required by the a priori power calculation, the relatively small cohort limits the generalizability of our findings and precludes robust subgroup analyses. Larger multicenter studies are therefore needed to validate these observations.

Third, the absence of a control group treated with medical therapy alone limits the ability to attribute the observed structural changes exclusively to CA. Although CA is currently the preferred rhythm-control strategy for appropriately selected patients with AF, and treatment variability was minimized by standardized antiarrhythmic therapy, a causal relationship cannot be definitively established.

Fourth, the follow-up duration of six months may not fully capture the long-term extent and durability of cardiac remodeling, as structural changes may persist beyond this period. Longer follow-up studies are therefore required to determine whether the observed imaging improvements persist and translate into clinically meaningful outcomes.

Finally, changes in body weight during follow-up were not systematically recorded and therefore could not be accounted for. Future studies comparing different CA modalities and evaluating associations between structural remodeling and long-term clinical outcomes, including AF recurrence, heart failure hospitalization, and quality of life, are warranted.

## 5. Conclusions

In conclusion, CA in patients with paroxysmal AF and HFpEF was associated with significant reductions in periatrial EAT volume and LVM, accompanied by favorable reverse cardiac remodeling as assessed by CMR. Correlation analyses further demonstrated that greater reductions in periatrial EAT volume were associated with more pronounced structural cardiac remodeling, supporting a close association between periatrial EAT regression and myocardial recovery after CA. Although AF burden was not directly quantified, the observed findings are consistent with the concept that a reduction in arrhythmia burden may help reverse cardiac remodeling in this population. Taken together, these findings provide new evidence that CA is associated with concurrent periatrial EAT regression and reverse cardiac remodeling in patients with paroxysmal AF and HFpEF. Larger prospective studies with longer follow-up, continuous rhythm monitoring for AF burden assessment, and comprehensive evaluation of clinical outcomes are warranted to confirm these observations and further elucidate the mechanistic links between AF burden, EAT, and cardiac remodeling.

## Figures and Tables

**Table 1:** Patients’ characteristics.

Clinical Characteristics	Mean and Standard Deviation
Age (years)	60.5 ± 10.4
Sex (male)	74.4%
Body height (cm)	177.7 ± 10.2
Body weight (kg)	89.3 ± 14.5
BMI (kg/m^2^)	28.3 ± 4.4
Average blood pressure at admission (systolic/diastolic in mmHg)	124/77 ± 9/6
NYHA class at admission	2.0 ± 0.2
H2FPEF score	7.0 ± 1.0
**Transthoracic echocardiographic parameters at enrollment**	**Mean and standard deviation**
LVEF (2D) (%)	59.5 ± 4.3
Left atrial volume index (mL/m^2^)	40.2 ± 5.7
E/e′ average	11.8 ± 1.9
Systolic pulmonary artery pressure (mmHg)	38.8 ± 3.3
**Comorbidities**	**Frequency (%)**
Arterial hypertension	46.5
Hyperlipidemia	65.1
Obesity (BMI > 30 kg/m^2^)	49.8
Overweight (BMI 25–30 kg/m^2^)	46.2
Prediabetes	20.9
**Antiarrhythmic therapy**	**Frequency (%)**
Propafenone	34.9
Propafenone + beta-blocker	65.1
**Laboratory parameter**	**Mean and standard deviation**
Hemoglobin (g/L)	128.3 ± 3.7
Creatinine (µmol/L)	99.2 ± 16.5
Estimated glomerular filtration rate (mL/min/1.73 m^2^)	59.6 ± 5.1
Serum glucose (mmol/L)	6.3 ± 0.8
HbA1c (%)	6.0 ± 0.1
C-reactive protein (mg/L)	4.0 ± 2.2
Low density lipoprotein cholesterol (mmol/L)	2.4 ± 0.3
Thyroid-stimulating hormone (mIU/mL)	1.2 ± 0.3
NT-proBNP (pg/mL)	180.1 ± 58.9

Note: BMI—body mass index; NYHA—New York Heart Association.

**Table 2:** Impact of CA on primary outcomes—periatrial EAT and LVM.

Parameter	Baseline	After 6 Months of Follow-Up	*p*-Value
EAT (mL)	28.8 ± 6.3	20.3 ± 5.9	<0.001
LVM (g/m^2^)	102.0 (95–115)	97.0 (89–104)	0.003

Note: EAT—epicardial adipose tissue; LVM—left ventricular mass.

**Table 3:** Impact of CA on cardiac remodeling (exploratory endpoints).

Parameter	Baseline	After 6 Months of Follow-Up	*p*-Value	q-Value (Adjusted *p*-Value)
LAA (cm^2^)	32.0 (29–36)	29.0 (26–31)	0.010	0.021
LVEDV (mL/m^2^)	79.0 (73–87)	77.0 (69–84)	0.037	0.038
LVESV (mL/m^2^)	36.0 (31–44)	32.0 (27–35)	0.011	0.021
LVSV (mL)	48.0 (42–54)	51.0 (46–58)	0.025	0.031
RAA (cm^2^)	28.0 (25–33)	28.0 (24–31)	0.990	0.990
RVEDV (mL/m^2^)	77.0 (70–87)	76.0 (68–83)	0.098	0.100
RVESV (mL/m^2^)	38.0 (35–45)	36.0 (32–43)	0.021	0.039
RVSV (mL)	48.0 (39–50)	49.0 (45–55)	0.039	0.064

Note: LAA—left atrial area; LVEDV—left ventricular end-diastolic volume; LVESV—left ventricular end-systolic volume; LVSV—left ventricular stroke volume; RAA—right atrial area; RVEDV—right ventricular end-diastolic volume; RVESV—right ventricular end-systolic volume; RVSV—right ventricular stroke volume.

**Table 4:** Correlations between changes in periatrial EAT volume and cardiac remodeling parameters after CA.

Parameter	Baseline	After 6 Months of Follow-Up	Change (Δ)	Spearman’s ρ (vs. ΔEAT)	*p*-Value *
EAT (mL)	28.8 ± 6.3	20.3 ± 5.9	−8.5 ± 2.1	—	—
LVM (g/m^2^)	102.0 (95–115)	97.0 (89–104)	−5.0 (−11 to −1)	+0.48	<0.001
LAA (cm^2^)	32.0 (29–36)	29.0 (26–31)	−3.0 (−6 to −1)	+0.44	0.003
LVEDV (mL/m^2^)	79.0 (73–87)	77.0 (69–84)	−2.0 (−6 to +1)	+0.31	0.042
LVESV (mL/m^2^)	36.0 (31–44)	32.0 (27–35)	−4.0 (−9 to 0)	+0.39	0.010
LVSV (mL)	48.0 (42–54)	51.0 (46–58)	+3.0 (0 to +7)	−0.41	0.006
RAA (cm^2^)	28.0 (25–33)	28.0 (24–31)	0.0 (−3 to +2)	+0.12	0.440
RVEDV (mL/m^2^)	77.0 (70–87)	76.0 (68–83)	−1.0 (−5 to +2)	+0.22	0.158
RVESV (mL/m^2^)	38.0 (35–45)	36.0 (32–43)	−2.0 (−5 to +1)	+0.25	0.105
RVSV (mL)	48.0 (39–50)	51.0 (46–58)	+3.0 (0 to +6)	−0.29	0.060

Note: * *p* < 0.05 significant, *p* < 0.001 highly significant. EAT—epicardial adipose tissue; LVM—left ventricular mass; LAA—left atrial area; LVEDV—left ventricular end-diastolic volume; LVESV—left ventricular end-systolic volume; LVSV—left ventricular stroke volume; RAA—right atrial area; RVEDV—right ventricular end-diastolic volume; RVESV—right ventricular end-systolic volume; RVSV—right ventricular stroke volume.

## Data Availability

The data that support the findings of this study are available from the corresponding author upon a reasonable request. The data are not publicly available due to ethical concerns.

## Abbreviations

The following abbreviations are used in this manuscript:

AF	Atrial fibrillation
EAT	Epicardial adipose tissue
CA	Catheter ablation
PVI	Pulmonary vein isolation
HFpEF	Heart failure with preserved ejection fraction
HFrEF	Heart failure with reduced ejection fraction
CMR	Cardiac magnetic resonance
LV	Left ventricle
LVEF	Left ventricular ejection fraction
LVEDV	Left ventricular end-diastolic volume
LVESV	Left ventricular end-systolic volume
LVSV	Left ventricular stroke volume
LVM	Left ventricular mass
RVEDV	Right ventricular end-diastolic volume
RVESV	Right ventricular end-systolic volume
RVSV	Right ventricular stroke volume
LAA	Left atrial area
RAA	Right atrial area
BMI	Body mass index
bSSFP	Balanced steady-state free precession
